# Climate change and the ash dieback crisis

**DOI:** 10.1038/srep35303

**Published:** 2016-10-14

**Authors:** Eric Goberville, Nina-Coralie Hautekèete, Richard R. Kirby, Yves Piquot, Christophe Luczak, Grégory Beaugrand

**Affiliations:** 1 Univ. Lille, CNRS, UMR 8198, Evo-Eco-Paleo, F-59 000 Lille, France; 2 CNRS, Univ. Lille, Univ. Littoral Côte d’Opale, UMR 8187, LOG, Laboratoire d’Océanologie et de Géosciences, F-62 930 Wimereux, France; 3 Sir Alister Hardy Foundation for Ocean Science, The Laboratory, Citadel Hill, Plymouth PL1 2PB, UK; 4 Marine Biological Association, Citadel Hill, The Hoe, Plymouth PL1 2PB, UK; 5 Université d’Artois, ESPE, Centre de Gravelines, 40 rue Victor Hugo - BP 129, 59820 Gravelines, France

## Abstract

Beyond the direct influence of climate change on species distribution and phenology, indirect effects may also arise from perturbations in species interactions. Infectious diseases are strong biotic forces that can precipitate population declines and lead to biodiversity loss. It has been shown in forest ecosystems worldwide that at least 10% of trees are vulnerable to extinction and pathogens are increasingly implicated. In Europe, the emerging ash dieback disease caused by the fungus *Hymenoscyphus fraxineus,* commonly called *Chalara fraxinea,* is causing a severe mortality of common ash trees (*Fraxinus excelsior*); this is raising concerns for the persistence of this widespread tree, which is both a key component of forest ecosystems and economically important for timber production. Here, we show how the pathogen and climate change may interact to affect the future spatial distribution of the common ash. Using two presence-only models, seven General Circulation Models and four emission scenarios, we show that climate change, by affecting the host and the pathogen separately, may uncouple their spatial distribution to create a mismatch in species interaction and so a lowering of disease transmission. Consequently, as climate change expands the ranges of both species polewards it may alleviate the ash dieback crisis in southern and occidental regions at the same time.

Disease alters ecosystems and can trigger biodiversity loss^1^,^2^. Recently, the frequency and magnitude of disease has been shown to be affected by climate change and related extreme events^3^,^4^, and forest ecosystems are no exception^1^,^5^. For example, in North Africa, the severe mortality of Atlas cedar (*Cedrus atlantica*) was brought about by a combination of a period of exceptional drought accompanied by fungal and insect injuries^5^. At least 10% of the world’s tree species are now vulnerable to disease, decline or extinction and pathogens are increasingly implicated^6^. Emerging infectious diseases are not a new concern^7^ but all evidence points towards an increase in the amount of disease and extinction events driven by fungi^7^,^8^.

In Europe, the common ash is currently threatened across most of its distributional range by a new lethal disease known as ‘Chalara dieback of ash’ that is caused by the ascomycete *Hymenoscyphus fraxineus*^9^. Ash dieback disease affects trees at various ages^6^,^10^ and, after first reports in Poland in the early 1990s, it has spread rapidly across Central, Northern and Western Europe^10^,^11^,^12^. Although forest managers might be inclined to remove infected trees^13^ or to abandon common ash planting, the economic value (e.g. common ash is estimated to make £22m/year in the United Kingdom^14^) and ecological role^10^,^11^ of this species argue for optimising tree reintroduction programs^10^,^11^. A thorough understanding of the environmental factors that might influence the geographical spread of the disease is therefore important. Here, we examine the potential influence of global climate change upon both the spatial distribution of common ash and its pathogen, and upon how these two species may interact in future.

## Results and Discussion

**Figure 1:**
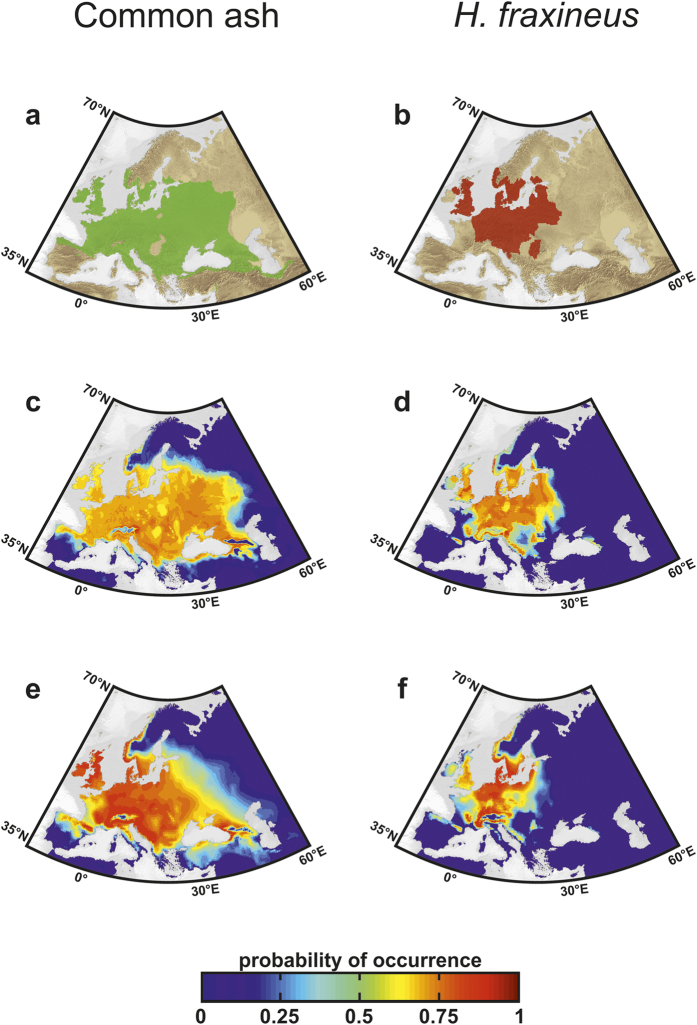
Observed and modelled spatial distributions of common ash and its pathogen. Observed distribution of (**a**) common ash (1950–2008) and (**b**) *H. fraxineus* (1992–2009) and modelled spatial distribution (as probability of occurrence) of (**c,e**) common ash and (**d**,**f**) *H. fraxineus* calculated from (**c**,**d**) MaxEnt and (**e**,**f**) NPPEN. Maps were produced using ArcGIS software v.10 (Environmental Systems Research Institute, Redlands, California, USA; http://www.esri.com/).

Common ash is widely distributed in Europe, ranging from the Atlantic coast to the Volga River in Russia (Fig. 1a); its northern limit occurs at ~64°N in Norway and its southern boundary is located at ~37°N and the environmental requirements of the species are well-known^15^,^16^. To estimate the spatial distribution of common ash (Fig. 1a) and *H. fraxineus* (Fig. 1b), we selected presence-only methods and applied a Species Distribution Model (SDM), the Maximum Entropy algorithm (MaxEnt^17^), and an Ecological Niche Model (ENM), the Non-Parametric-Probabilistic-Ecological-Niche model (NPPEN^18^). The bioclimatic parameters were derived from the Climate Research Unit dataset (Supplementary Table S1). The consistency between simulated contemporary occurrences and species observations (Fig. 1) was assessed by calculating the area under the curve (AUC) and the true skill statistic (TSS). Our results revealed that both models showed high ability to reproduce the overall species distributions; AUC values were as high as 0.96 and TSS values ranged from 0.83 and 0.92 (Supplementary Table S2). Although MaxEnt reproduces the contemporary spatial distribution of the host and its pathogen slightly better, the two models showed comparable estimates of contemporary geographical extent (Supplementary Table S2). Our projections (Fig. 1c,e) confirmed that the spatial distribution of common ash is constrained by aversion to cold winters and hot dry summers^10^,^15^. The European distribution of the pathogen was reproduced well by the two models and no occurrence of *H. fraxineus* was predicted in regions where ash was not reported (Fig. 1b,d,f).

**Figure 2:**
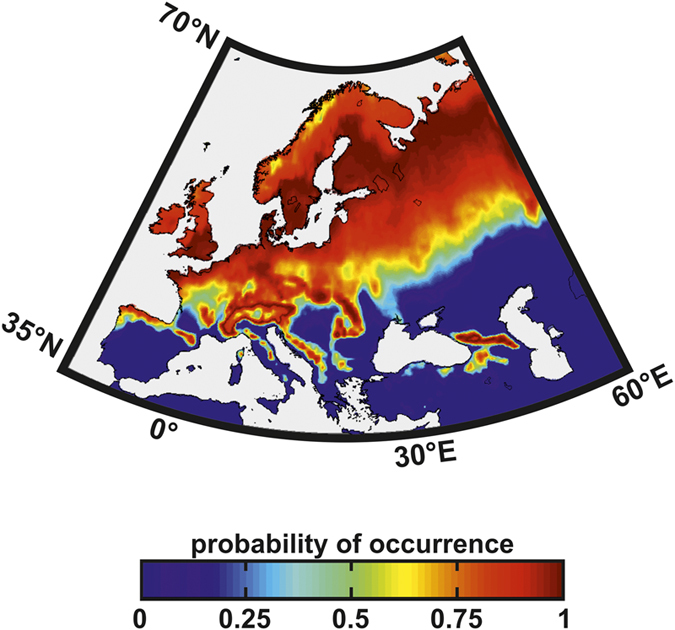
Modelled spatial distributions of *Hymenoscyphus fraxineus*. Modelled spatial distribution (as probability of occurrence) of *H. fraxineus* calculated from a model based on the physiological tolerances of *H. fraxineus* to temperature using the limits defined by Hauptman *et al.*^23^ (see Supplementary Figure S3). Map was produced using Matlab R2015b (http://www.mathworks.com).

Recent phylogenetic analyses of *H. fraxineus* suggest an Asian origin for this ascomycete, however^9^,^19^. As the fungus has only been reported in a few places in Japan, Korea, China and Far East Russia (Supplementary Figure S1) we did not consider its native range to determine its ecological niche but its European invasive range (Fig. 1b). Among the environmental factors that most seriously affect the initiation and development of diseases, it is well documented that temperature is critical^1^,^5^,^20^,^21^. *Hymenoscyphus fraxineus* is a cold-tolerant-organism, with an optimal temperature at around 20–22 °C and no fungal growth takes place above 28 °C (refs 22, 23, 24). Using a model based on the physiological tolerances of *H. fraxineus* to temperature (Supplementary Figure S3), we evaluated whether all suitable thermal habitats in Europe have already been colonised by the pathogen. This analysis revealed that the pathogen is close to equilibrium with its climate in southern Europe. Although the pathogen might be more widespread than observed in Scandinavia and in Baltic States, the thermal niche of *H. fraxineus* (Fig. 2), occurrence data (Fig. 1b) and simulations calculated with MaxEnt and NPPEN (Fig. 1d,f) suggested unsuitable warm climatic conditions in western and southern regions^23^,^25^, which is also corroborated by multi-model and spatially explicit approaches^12^.

**Figure 3:**
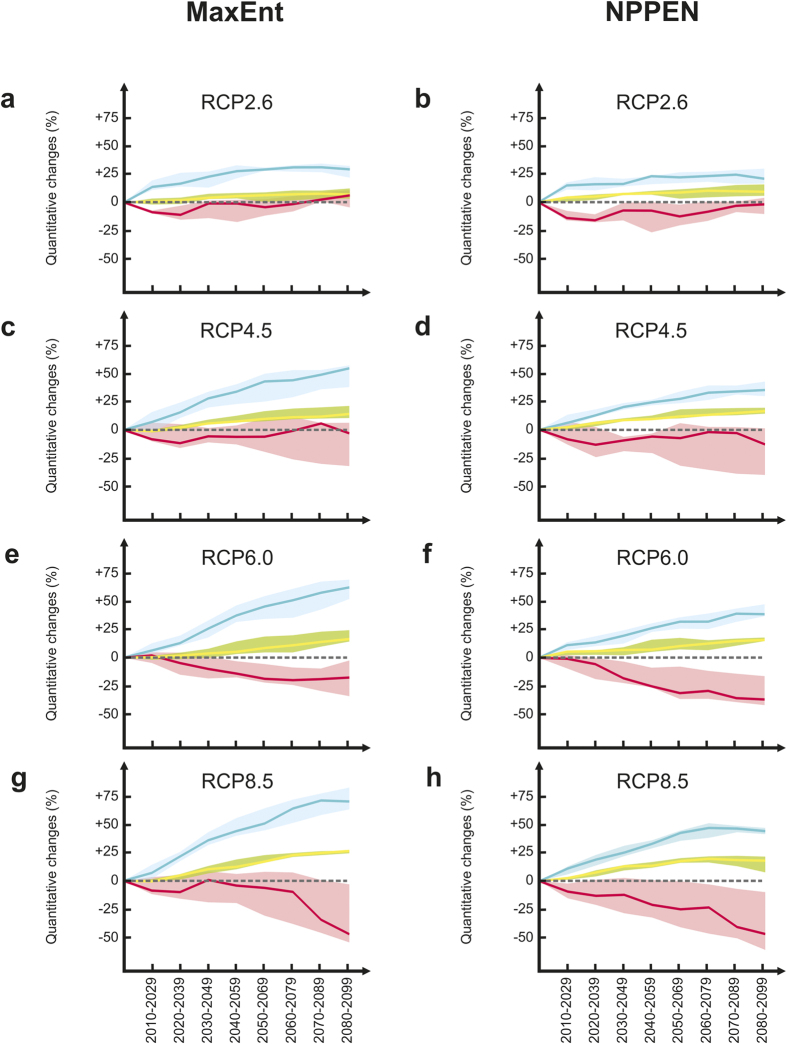
Expected long-term changes in the spatial distribution of common ash and its pathogen using MaxEnt and NPPEN. Long-term quantitative changes (median and both first and third quartiles as shading) relative to 1992–2009 for *H. fraxineus* (in red), and common ash without (in green) and with (in blue) the interactive effect of *H. fraxineus* using (**a**,**c**,**e**,**g**) MaxEnt and (**b**,**d**,**f**,**h**) NPPEN for scenarios (**a**,**b**) RCP2.6, (**c,d**) RCP4.5, (**e**,**f**) RCP6.0 and (**g**,**h**) RCP8.5.

Change in the spatial distribution of common ash and its pathogen under future climates was investigated using ‘Representative Concentration Pathways’ (RCPs) scenarios^26^. The pathogen-host interaction was modelled by constraining the distribution of common ash by the presence of *H. fraxineus*, a common approach to incorporate biotic interactions at large spatial extents^27^,^28^. Both models revealed a positive effect of climate upon the geographical extent of common ash and this was also frequently combined with a reduction in pathogen prevalence (Fig. 3) in relation to the different levels of warming (Supplementary Figure S5). The percentage of increase in the geographical extent of common ash also depended on the magnitude of warming. For Scenario RCP2.6, the spatial area covered by common ash (excluding the fungus) was predicted to increase from 1 to 10% over the 21^st^ century (Fig. 3a,b). The coverage of the pathogen rose after an initial decrease although remaining frequently negative (from −16% to +6%). When the interactive effect between the tree and its pathogen was taken into account, the combined effect translated into an increase in the geographical extent of the tree ranging from 15 to 30% (Fig. 3a,b). For Scenario RCP4.5, the climatic effect on common ash was higher in magnitude (from 2–17%) whilst changes in *H. fraxineus* were similar to those expected with RCP2.6 (from −14% to +6%; Fig. 3c,d). Consequently, both models projected an increase in common ash when the influence of the fungus was considered (>50% with MaxEnt and >35% with NPPEN for 2080–2099). For Scenario RCP6.0, the influence on the tree was similar to that observed for a lower level of warming but strongly negative for the pathogen (Fig. 3e,f). From 2050, a reduction between 20–37% of *H. fraxineus* was observed. When the interactive effect of the pathogen was included, both models projected an increase greater than 40% in the geographical extent of common ash at the end of the century (Fig. 3e,f). For Scenario RCP8.5, the climatic effect on the tree remained highly positive (from 17–26% from 2050) although a plateau was observed with NPPEN from 2060 (Fig. 3g,h). Although occurring later with MaxEnt, the two ecological models projected a pronounced reduction in the pathogen (~50% for the last decade) and revealed the major impact of high temperature on the viability and growth of *H. fraxineus*^22^,^23^,^24^. When the pathogenic influence was considered, changes in common ash varied in both direction and magnitude, and plateaued from 2060 with NPPEN. Consequently, the maximum gain was obtained with MaxEnt for the last decades (>70% compared to >45% with NPPEN).

**Figure 4:**
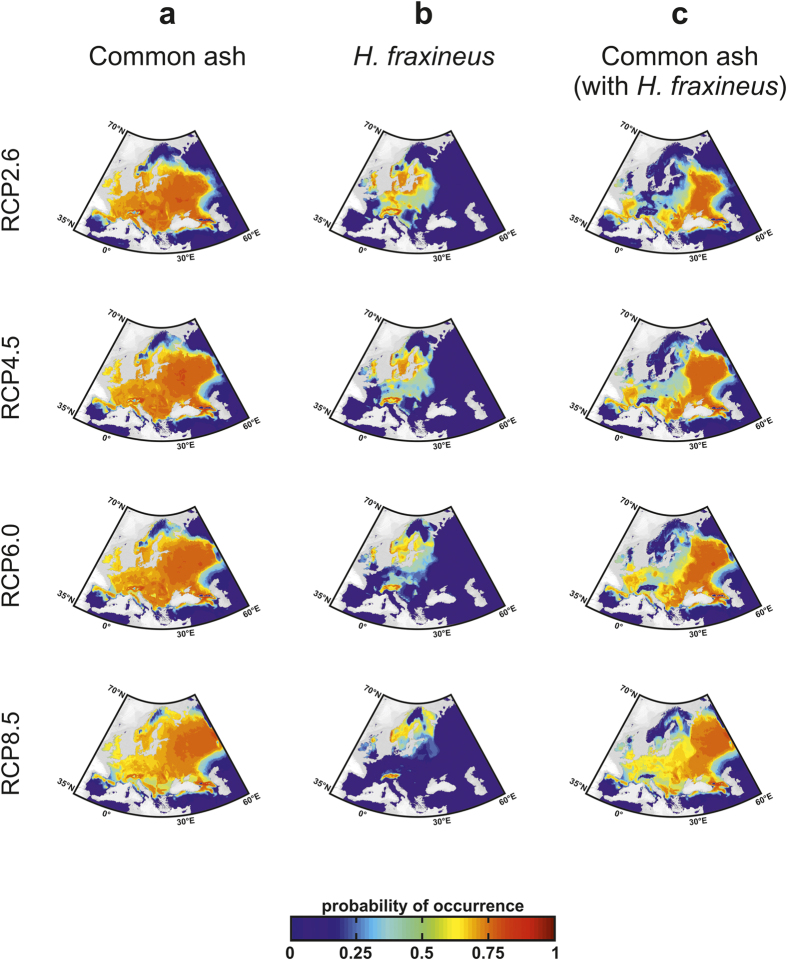
Expected future spatial distribution of common ash and its pathogen calculated from MaxEnt. Projections of the averaged probability of occurrence of (**a**) common ash, (**b**) *H. fraxineus* and (**c**) common ash with consideration of the interactive effect of the pathogen (termed “common ash with *H. fraxineus”*) for 2080–2099 using scenarios RCP2.6, RCP4.5, RCP6.0 and RCP8.5. Maps were produced using ArcGIS software v.10 (Environmental Systems Research Institute, Redlands, California, USA; http://www.esri.com/).

**Figure 5:**
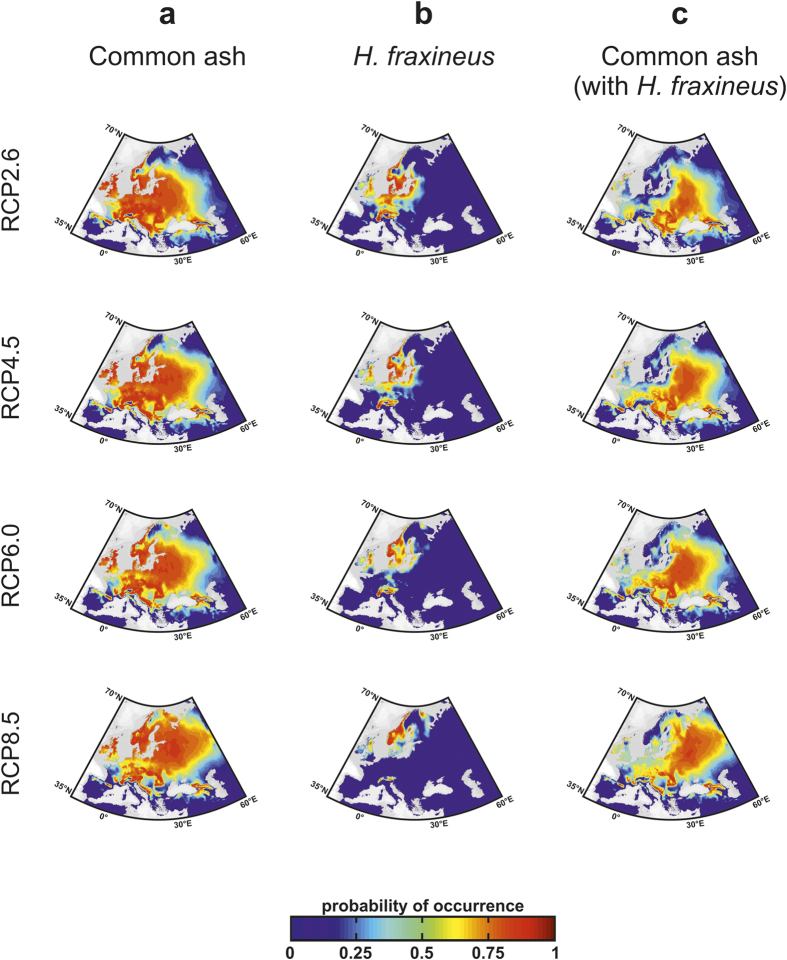
Expected future spatial distribution of common ash and its pathogen calculated from NPPEN. Projections of the averaged probability of occurrence of (**a**) common ash, (**b**) *H. fraxineus* and (**c**) common ash with *H. fraxineus* for 2080–2099 using scenarios RCP2.6, RCP4.5, RCP6.0 and RCP8.5. Maps were produced using ArcGIS software v.10 (Environmental Systems Research Institute, Redlands, California, USA; http://www.esri.com/).

We investigated changes in the spatial distribution of the host and its pathogen associated with global climate change by averaging species projections resulting from the 7 GCMs for each RCP scenario for the period 2080–2099 (Figs 4 and 5). Spatial changes in species distribution were drastic for *H. fraxineus* for both ecological models even for small degrees of warming (Fig. 4b *versus* Fig. 5b); in particular, the pathogen was projected to disappear from France especially because of unsuitable warm climatic conditions which alter the spread of infectious diseases^1^,^5^,^20^,^21^ (Supplementary Figure S5). For Scenarios RCP2.6 and RCP4.5, both direct and indirect effects of climate change fragmented the spatial range of common ash (Figs 4c and 5c). When global warming was pronounced (RCP6.0 and RCP8.5) both MaxEnt and NPPEN indicated there would be a major poleward movement of the pathogen, with populations only persisting in Scandinavia and the Baltics. The occidental common ash populations were only slightly affected when warming became too intense (Scenarios RCP6.0 and RCP8.5), especially for projections based on NPPEN (Fig. 4a *versus* Fig. 5a). Although the direct effect of climate on common ash was negative in occidental and southern European countries (e.g. England, France or the Balkans), the virulence of the fungus is also expected to decrease in these areas. When host populations decline and fragment, pathogens with density-dependent transmission face a lowered chance of infection^29^, and while the strength of this positive feedback is difficult to evaluate in our study, it could reduce infection rates at the southern edge of common ash distribution but exacerbate infection at the centre of the spatial distribution of the tree^29^.

We realise that phenomenological models have a number of limitations. First, to model the ecological niche of both species, we had to assume that the contemporary distribution of common ash and its pathogen is in equilibrium with climate^30^. Although that assumption is likely to be correct for the pathogen, especially in southern Europe (Fig. 2), the longevity of adult trees inevitably means they will persist for a while after unsuitable climatic conditions have appeared^31^,^32^. Second, we also assumed unlimited dispersal, which is unlikely to be the case for common ash even though dispersal distances up to 3 kilometres have been recorded^33^; here, the main consequence would be to anticipate too quickly the arrival of the tree to newly suitable areas. Third, our model does not consider either additional projected infestations (e.g. the Emerald Ash Borer^34^) or the effect of extreme climatic events that may affect existing populations strongly^4^. For example, the extreme heat wave and drought during summer 2003 triggered the collapse of Scot pine (*Pinus sylvestris*) populations in Southeastern France^5^. We also do not consider possible variation in the susceptibility of different ash genotypes to *H. fraxineus*^35^ and we assume ecological niche conservatism^36^, i.e. the niche shape remains unchanged over the study period. Unfortunately, phenomenological models cannot consider processes of host-pathogen coevolution that may influence our results^37^,^38^. Despite those limitations, our study investigates a large range of scenarios using two presence-only models (a SDM and an ENM), up to seven GCMs and four levels of warming and considers both direct and indirect (i.e. interactive effect of the pathogen) effects of climate change on common ash showing how climate change factors should at least be considered when planning ecosystem management.

Over the next decades, both climate change and disease are expected to affect forest ecosystems considerably^3^,^6^,^39^. Some diseases may be controlled by climate because both temperature and water availability govern pathogen development^10^,^11^, host susceptibility^10^,^11^ and thereby disease-spreading^1^. Here, our study also reveals that climate change, by affecting the host and the pathogen separately, may uncouple their spatial distributions, involving a mismatch in the species’ interaction^27^ with putative consequences for density-dependent transmission^29^. Consequently, although the direct effect of climate change upon common ash is expected to be negative in southern and occidental regions where warming might favour the extension of the Mediterranean ash *F. angustifolia*^15^, it may at the same time alleviate the ash dieback crisis as climate change expands the ranges of both species polewards^25^. Our study therefore reveals the complexity of biological pathways through which climate change may affect species and biotic interactions^38^,^39^,^40^. Distribution models could provide useful information to help design reintroduction programmes to better manage forest ecosystems^41^. While foresters are unwilling to invest in common ash planting, our results show that it is too early to abandon its exploitation. However, it remains challenging to better document host-pathogen interactions^1^,^3^,^40^ and a deeper knowledge of the infection process would allow us to refine modelling approaches^10^,^11^: landscape pathology would provide insights about dispersal ability of the fungus^42^, genomic sequencing procedures would help to identify the origin of the disease^19^. Initiatives are already ongoing to ensure the sustainability of common ash in Europe (e.g. the FRAXBACK COST action, Nornex) and will allow foresters to base conservation decisions not on instinctive reactions but instead on ecological scenarios^41^,^43^.

## Methods

### Biological Data

The distribution map of common ash (*Fraxinus excelsior*) was downloaded from the EUFORGEN (European Forest Genetic Resources Programme) website (Fig. 1a; http://www.euforgen.org/distribution_maps.html). Because no comprehensive database on the spatial distribution of *Hymenoscyphus fraxineus* was available, we digitalised and compiled occurrence data from different recent maps found in the literature^10^,^44^,^45^ to produce the most up-to-date distribution of the fungus (Fig. 1b) (see Supplementary Information for details).

### Climatic Data

We used the gridded Climatic Research Unit (CRU) TS (time-series) 3.10.01 climatic datasets provided by the Climate Research Unit^46^ and obtained from the British Atmospheric Data Centre (BADC; http://badc.nerc.ac.uk/). From monthly averages of minimum/maximum temperature and precipitation data over the period 1901–2009, a set of bioclimatic parameters was calculated (Supplementary Table S1) by applying the procedure described by Ramirez-Villegas & Bueno-Cabrera^47^.

### Modelling Procedure

To estimate the spatial distribution of common ash and *H. fraxineus,* we selected two presence-only methods: the Maximum Entropy (MaxEnt) algorithm^17^ and the Non-Parametric-Probabilistic-Ecological-Niche (NPPEN) model^18^ (see SI Materials and Methods for further details). We selected the most important bioclimatic parameters based on their relative contribution to the estimation of the spatial distribution of common ash and its pathogen (i.e. variables with a contribution of at least 5%) and possible collinearity between predictors was evaluated by calculating pairwise correlation coefficients (see Supplementary Information, Supplementary Table S1 and Supplementary Figure S2). The following two sets of parameters were retained: (*i*) precipitation of the driest quarter, annual mean temperature, mean temperature of the coldest quarter and precipitation of the warmest quarter for common ash and (*ii*) annual temperature range, precipitation of the warmest quarter, mean temperature of the coldest quarter and maximum temperature of the warmest month for *H. fraxineus*. The assumption of equilibrium between the fungus and climate was tested by performing a model based on the physiological tolerances of *H. fraxineus* to temperature (Supplementary Figure S3) using the limits determined experimentally by Hauptman and colleagues (see Table 2, Figs 1 and 2 in their publication^23^). We then estimated the direct influence of climate change on the spatial distribution of common ash and *H. fraxineus* and its indirect effects through pathogen-host interaction for eight 20-year periods (from 2010–2029 to 2080–2099) using the “Representative Concentration Pathways” (RCPs) climate scenarios, from the fifth phase of the Coupled Model Intercomparison Project (CMIP5^26^). Seven different General Circulation Models (GCMs; see Supplementary Table S3 for basic information) and the four greenhouse gas concentration emission scenarios were considered: RCP2.6, RCP4.5, RCP6.0 and RCP8.5 (from the most optimistic to the most pessimistic).

### Pathogen-Host Interaction

The pathogen-host interaction (termed ‘common ash with *H. fraxineus’*) was modelled by constraining the distribution of common ash by the presence of *H. fraxineus*, i.e. by calculating the differences in probability of occurrence between common ash and *H. fraxineus* in each geographical cell. This method is comparable to the one implemented by Schweiger and colleagues^27^,^48^ to assess butterfly distributions taking into account the distribution of their larval host plants. Such an approach minimises potential modelling issues, especially when two species responds in a similar way to bioclimatic parameters^28^.

### Quantification of Changes in the Geographical Extent of Species

To quantify changes in the area covered by the common ash and its pathogen, we transformed probability of occurrence values into binary predictions (i.e. presence/absence) using decision thresholds^49^ (Supplementary Table S2). For the period 1992–2009 which corresponds to the observations of the fungus and each future time period, we calculated the area covered by the tree, the pathogen and the coverage of the common ash with *H. fraxineus*. For each presence-only model, each GCM and each RCP scenario, changes in the geographical extent of species relative to the period 1992–2009 were estimated in percentage. The same procedure was performed to evaluate changes in the geographical extent of the thermal niche of *H. fraxineus* (Supplementary Figure S5). For each simulation, the median, first and third quartiles were calculated to show the variability among GCMs (Fig. 3; Supplementary Table S4).

## Additional Information

**How to cite this article**: Goberville, E. *et al.* Climate change and the ash dieback crisis. *Sci. Rep.* **6**, 35303; doi: 10.1038/srep35303 (2016).

## Supplementary Material

Supplementary Information
