# Supplementary material for: Climate change and the ash dieback crisis
Source: Sci Rep. 2016 Oct 14;6:35303. doi: 10.1038/srep35303 (PMC5064362; doi:10.1038/srep35303)
Supplement: Supplementary Information [file srep35303-s1.pdf]

Climate change and the ash dieback crisis

Eric Goberville[1,2,3,*], Nina-Coralie Hautekèete[1], Richard R Kirby[4], Yves Piquot[1], Christophe Luczak[2,5], and Grégory Beaugrand[2,3,*]

[1]Univ. Lille, CNRS, UMR 8198, Eco-Evo-Paleo, F-59 000 Lille, France

[2]CNRS, Univ. Lille, Univ. Littoral Côte d'Opale, UMR 8187, LOG, Laboratoire d'Océanologie et de Géosciences, F-62 930 Wimereux, France

[3]Sir Alister Hardy Foundation for Ocean Science, The Laboratory, Citadel Hill, Plymouth PL1 2PB, UK

[4]Marine Biological Association, Citadel Hill, The Hoe, Plymouth PL1 2PB, UK

[5]Université d'Artois, ESPE, Centre de Gravelines, 40 rue Victor Hugo - BP 129, 59820 Gravelines, France

[*]eric.goberville@univ-lille1.fr or gregory.beaugrand@univ-lille1.fr

Supplementary Information

Submitted to Scientific Reports

SUPPLEMENTARY METHODS

Biological data

Distribution maps of common ash (from EUFORGEN; http://www.euforgen.org/distribution_maps.html) and *H. fraxineus* (after digitalisation from different recent maps found in the literature[1-3]) were converted to raster format using ArcGIS software v.10 (Environmental Systems Research Institute, Redlands, California, USA). Occurrence data were then aggregated on a 0.5 x 0.5 spatial grid that corresponded to the gridded climatic data.

The Asian distribution of *H. fraxineus* (Supplementary Fig. S1) was compiled from the European and Mediterranean Plant Protection Organization (EPPO) Global Database (https://gd.eppo.int/) using the provided references for more detailed locations[4-7].

Climatic data

We used the gridded Climatic Research Unit (CRU) TS (time-series) 3.10.01 climatic datasets provided by the Climate Research Unit[8] and obtained from the British Atmospheric Data Centre (BADC; http://badc.nerc.ac.uk/). Datasets are based on an archive of monthly climate parameters provided by more than 4000 weather stations distributed around the world and are calculated on 0.5 x 0.5 degree grids[8]. CRU TS3.10.01 includes monthly averages of minimum/maximum temperature and precipitation data for the period 1901-2009.

To model the spatial distribution of the two species, climate data were averaged (*i*) for the period 1950-2008 (corresponding to the EUFORGEN map) for common ash and (*ii*) the period 1992-2009 (in accordance with observations found in the literature) for *H. fraxineus*. From minimum/maximum temperature and precipitation data, a set of 19 bioclimatic parameters (Supplementary Table S1) revealing seasonal means and extremes of precipitation and temperature was calculated, applying the procedure described by Ramirez-Villegas & Bueno-Cabrera[9]. These bioclimatic variables could be more strongly related to species distributions than annual averages[10]. Because of their link with both the physiology and growth of terrestrial species, they are biologically significant for characterising species range[11,12,13]. These bioclimatic parameters are considered as the basic bioclimatic dataset for niche-based modelling[14].

Presence-only modelling methods

The MaxEnt model

The Maximum Entropy (MaxEnt[15]) model based on the maximum entropy, is one of the most commonly used Species Distribution Model (SDM) for modelling species distributions from presence-only species data. As MaxEnt has been extensively applied over the past decade[16] and exhaustively documented[15,16,17,18], we refer the reader to the literature for a thorough description of the model. We utilised the Maxent software version 3.3.3k using a maximum of 500 iterations, the logistic output and standard settings[16]. Random test percentage was set at 30. The regularisation multiplier was set to 1 as a good compromise to limit both over- and underfitting[19]. Lastly, we generated 10,000 background points chosen uniformly at random from the whole area[18]. For each species, 4 replicates were run and the mean probability of occurrence was calculated.

The Non-Parametric Probabilistic Ecological Niche model (NPPEN).

The NPPEN model[20] is an Ecological Niche Model (ENM) specifically designed to be applied on presence-only data. This technique allows the modelling of the ecological niche of a species and the mapping of its spatial distribution by calculating its probabilities of occurrence. The first step consists in constructing a reference matrix with environmental data corresponding to the presence records. Following a procedure similar to the one performed in the program RASTERIZ included in the GARP modelling system[21], the reference matrix is homogenised to remove as far as possible the inaccurate reporting of occurrence records[22]. It results a multidimensional matrix with each of the dimension reflecting an environmental factor. In a second step, the Mahalanobis generalised distance[23] is calculated between the observations and the homogenised reference matrix. The use of the Mahalanobis distance presents the double advantage of taking into account the correlation between variables[24] and being independent of the scales of the descriptors[25]. In the third step, the model calculates the probability of each grid point to belong to the reference matrix by using a simplified version of the Multiple Response Permutation Procedure (MRPP[26]). Finally, the probability of species occurrence are mapped in the geographical space. More information on the model is available in the literature[20,22,27,28].

Model accuracy

To evaluate the consistency between simulated and observed spatial distributions, we used the area under the relative characteristic curve (AUC[29]) and the true skill statistic (TSS[30]) procedures. In the case of a presence-only model, the AUC value describes the probability that the model scores a random presence site higher than a random background site[31] and the value varies[32] between 0.5 (for random performance) and 1 (a perfect fit). The TSS value compares the number of correct predictions, minus predictions attributable to random guessing, to that of a hypothetical set of perfect predictions[30]. TSS ranges from -1 to +1, where +1 indicates perfect agreement between modelled and observed distributions. A value close to zero or less reveals a poor predictive ability.

We used a data-splitting procedure to evaluate model accuracy, by randomly selecting 70% of data to run our models and 30% of the remaining data for evaluation[33]. Background locations were chosen randomly 500 times in the whole spatial domain to calculate both the mean and the standard deviation of the measures of performance (Supplementary Table S2).

While the selection of a suitable procedure to assess model accuracy remains widely discussed[34], these two commonly used measures of the performance of modelling procedures have the advantage of being independent of prevalence (i.e. frequency of presence records in the whole spatial domain[30,35]).

Selection of the best combination of bioclimatic variables

Because no variable selection procedure exists for the NPPEN model and to carry out consistent comparisons between the two presence-only models (i.e. by using the same set of bioclimatic variables), we estimated, for both species, the relative contributions of each of the 19 bioclimatic variables (Supplementary Table S1) using the MaxEnt software package. For common ash and its pathogen, we first performed 10 models runs with a random alternation of the training (70% of data) and validation (the remaining 30%) datasets and we ranked the 19 bioclimatic variables on

the basis of their averaged relative contribution (Supplementary Table S1). By means of the AUC procedure to test agreement between observations and contemporary projections (see '***Model accuracy***'), we also determined the influence of considering an additional predictor following an incremental approach from only one bioclimatic variable used to build the model to the consideration of the whole bioclimatic dataset. We then decided to handpick only the variables with a contribution of at least 5%, i.e. the first four bioclimatic variables which mostly contribute to the spatial distribution of common ash and its pathogen. Note that for both species, the model accuracy (AUC value) did not increase by more than 1% when a fifth parameter was considered (Supplementary Table S1).

Doing so, the following two sets of bioclimatic parameters were retained (Supplementary Table S1; a quarter represents a period of three months):

- Common ash: (*i*) Precipitation of the driest quarter, (*ii*) Annual mean temperature, (*iii*) Mean temperature of the coldest quarter, (*iv*) Precipitation of the warmest quarter.
- *Hymenoscyphus fraxineus*: (*i*) Annual temperature range, (*ii*) Precipitation of warmest quarter, (*iii*) Mean temperature of the coldest quarter, (*iv*) Maximum temperature of the warmest month.

For each set of bioclimatic parameters, we investigated potential collinearity among variables using pairwise correlation coefficients (Supplementary Figure S2). For *H. fraxineus*, all correlations were either under or close to the threshold values commonly used (e.g. ref. 36 and references therein, refs. 37 and 38), indicating an absence of strong multicollinearity. Because of the high correlation detected between mean temperature of the coldest quarter and annual mean temperature (r=0.86; Supplementary Figure S2a) for the common ash dataset, we also calculated the variance inflation factors (VIFs) to quantify collinearity. No VIF value was greater than 10 (i.e. 3.4, 6.7, 7.0 and 3.1 for precipitation of the driest quarter, annual mean temperature, mean temperature of the coldest quarter, and precipitation of the warmest quarter; respectively), showing that the influence of multicollinearity was limited[37].

Although we recognise that additional parameters may also influence locally the spatial distribution of those species[39], the implementation of a large number of parameters into a model may increase the risk of multicollinearity[40].

Model based on the thermal tolerance of H. fraxineus

To handle the uncertainty related to a potential non-equilibrium of the pathogen with climate, we investigated whether all suitable thermal habitats in Europe have been invaded by the pathogen. We modelled the thermal niche of *H. fraxineus* (Supplementary Fig. S2) using the physiological limits determined experimentally by Hauptmann et al.[41] who has characterised the effect of temperature on *H. fraxineus* development and growth: the pathogen is a cold-tolerant-organism, with an optimal temperature at around 20-22°C and no fungal growth at 28°C and above[41,42,43]. To provide an assessment of the variability among isolates (i.e. the two different growth media and the four common ash saplings; see Table 2, Figures 1 and 2 in Hauptmann et al.[41]), the median and both first and third quartiles of the thermal niche have been considered in our model (Supplementary Fig. S2). The thermal niche of the species was then mapped in the geographical space (Figure 2).

Decision thresholds to quantify the geographical extent of species

To quantify the area covered by common ash and its pathogen, we calculated decision thresholds above which modelled distributions outputs are considered to represent species occurrence (Supplementary Table S2). For both species and each presence-only model, we calculated the value that minimises the distance between the ROC plot and the top left corner of the unit square[44]. This method, advocated when the influence of climate change on a species range is investigated[45,46], is known to improve the spatial prediction of species by removing false positive presence[45,46,47].

Estimation of the future bioclimatic parameters using data originated from General Circulation Models (GCMs)

We estimated the direct influence of climate change on the spatial distribution of common ash and *H. fraxineus* and its indirect effects through pathogen-host interaction using the 'Representative Concentration Pathways' (RCPs) climate scenarios[48], from the fifth phase of the Coupled Model Intercomparison Project (CMIP5). These RCPs have replaced Special Report on Emissions Scenarios (SRES) and provide a range of possible futures for the evolution of atmospheric composition[49,50]. Global climate simulations are based on greenhouse gas emission scenarios[48] corresponding to peaking radiative forcing of 3.0 $W.m^{-2}$ within the 21st century and declining afterwards to 2.6 $W.m^{-2}$ (RCP2.6), stabilisation of radiative forcing at 4.5 $W.m^{-2}$ (RCP4.5) and 6.0 $W.m^{-2}$ (RCP6.0) after the 21st century, and rising radiative forcing leading to 8.5 $W.m^{-2}$ at the end of 21st century (RCP8.5). Monthly time series of minimum/maximum temperature and precipitation for the period 1950-2100 from seven Global Circulation Models (GCMs) and the four RCPs scenarios were downloaded from the Earth System Grid Federation portal (ESGF; http://pcmdi9.llnl.gov/esgf-web-fe/). Basic information on each GCM is provided in Supplementary Table S3.

We first calculated climatologies in minimum/maximum temperature and precipitation for the two contemporary periods: 1950-2008 for common ash and 1992-2009 for *H. fraxineus*. For each species, we obtained 252 climatologies (7 GCMs x 3 variables x 12 months). To project the potential future species distributions, we calculated future climatic conditions for eight 20-year periods (i.e. 2010-2029, 2020-2039, 2030-2049, 2040-2059; 2050-2069, 2060-2079; 2070-2089 and 2080-2099). This procedure was applied for the seven GCMs and the four RCPs and for each month (twelve) of each period (eight), giving a total of 2400 future climatologies for each of the three parameters (i.e. minimum/maximum temperature and precipitation).

For each 20-year period, anomalies in minimum/maximum temperature and precipitation were computed for each month (i.e. difference between a given 20-year period and each of the two contemporary periods). Interpolation was applied to generate gridded data of monthly anomalies in minimum/maximum temperature and precipitation at the spatial resolution of 0.5° latitude x 0.5° longitude using the minimum curvature method from the Spatial and Geometric Analysis toolbox (SaGA; http://puddle.mit.edu/~glenn/kirill/saga.html). Although other interpolation methods exist[51,52], this procedure is known as suitable and computationally efficient to perform downscaling[53,54].

Interpolated anomalies were then added to the two contemporary climatologies in minimum/maximum temperature and precipitation (1950-2008 and 1992-2009 for common ash and

H. fraxineus, respectively) following the 'delta method' procedure provided by Ramirez-Villegas & Jarvis[55]. This method maintains the adequacy between GCM outputs and the CRU dataset. We then applied the procedure provided by Ramirez-Villegas & Bueno-Cabrera[9] to obtain the same sets of parameters than those retained to calculate the contemporary spatial distribution of the two species, but for future conditions.

Quantification of changes in the geographical extent of species

For the period 1992-2009 which corresponds to the observations of the fungus (see the section '***Biological data***') and the 8 future time periods, we calculated the area covered by the tree, the pathogen and the coverage of common ash with *H. fraxineus*. For the two presence-only models and the model based on the thermal tolerance of *H. fraxineus*, changes in the geographical extent of species (in percentage) relative to the period 1992-2009 were estimated for each of the seven GCMs and each level of warming (i.e. RCP scenario). To provide an assessment of the variability among GCMs, the median and both first and third quartiles were calculated (Fig. 3; Supplementary Table S4; Supplementary Fig. S4). Surface areas (in million km^2) were estimated using the areaquad function of Matlab, assuming the spherical ellipsoid of the Earth.

SUPPLEMENTARY FIGURE AND TABLES

Figure S1. Distribution map of the occurrence of *H. fraxineus* in Asia from the European and Mediterranean Plant Protection Organization (EPPO) Global Database (https://gd.eppo.int/). Maps were produced using ArcGIS software v.10 (Environmental Systems Research Institute, Redlands, California, USA; http://www.esri.com/).

Figure S2. Results from the pairwise correlation analysis. Correlation coefficients among pairs of bioclimatic parameters retained to model (a) common ash and (b) *H. fraxineus.* Histograms (in green) show the distribution of each parameter.

Figure S3. Thermal niche of *H. fraxineus*. Effect of temperature on *H. fraxineus* growth rates (in mm/day). The median (solid line) and both first and third quartiles (dotted lines) of the thermal niche provide an assessment of the variability among the different isolates considered in (see Table 2 in Hauptmann et al.[41]).

Figure S4. Expected long-term changes in the spatial distribution of *H. fraxineus* calculated from its thermal niche. Long-term quantitative changes (median and both first and third quartiles as shading) for *H. fraxineus* for scenarios (a) RCP2.6, (b) RCP4.5, (c) RCP6.0 and (d) RCP8.5.

Figure S5. Expected future spatial distribution of *H. fraxineus* calculated from its thermal niche. Projections of the averaged probability of occurrence of *H. fraxineus* for 2080-2099 using scenarios RCP2.6, RCP4.5, RCP6.0 and RCP8.5. Maps were produced using Matlab R2015b (http://www.mathworks.com).

Table S1. Selection of the best combination of bioclimatic parameters. Ranking of the bioclimatic parameters used in the study according to ascending model relative contribution (mean of 10 model runs) for (a) common ash and (b) *H. fraxineus*. AUC values quantify the gain in predictive accuracy of the MaxEnt model when the corresponding parameter is included in addition to the previous one

(from (1) to (19) predictors). For each species, the four first bioclimatic parameters (in bold) were retained for further analysis. Calculation performed with the MaxEnt software package.

Table S2. Predictive accuracy of the MaxEnt and NPPEN models. AUC and TSS values (average and standard deviation) for both species and each ecological model. Thresholds are the values above which modelled distributions outputs are considered to represent species occurrence. Contemporary geographical extents are estimated from the modelled spatial distributions of species for the period 1992-2009 after application of the decision thresholds.

Table S3. General Circulation Models (GCMs) used in this study. Detailed descriptions of each GCM can be found in the references cited in the Table.

Table S4. Expected quantitative changes in species distributions for each time period and a given level of warming. Median (bold), first and third quartiles (italics) of projected quantitative changes (calculated from the seven GCMs) in the area covered by common ash (excluding the fungus), *H. fraxineus* (excluding the tree) and common ash with consideration of the interactive effect of the pathogen (termed 'common ash with *H. fraxineus*') relative to the contemporary periods for scenarios RCP2.6, RCP4.5, RCP6.0 and RCP8.5 and the 8 time periods from 2010-2029 to 2080-2099 calculated from (A) MaxEnt and (B) NPPEN.

SUPPLEMENTARY REFERENCES

1. Bengtsson, S.B.K. Dieback of *Fraxinus excelsior*, biology of ash dieback and genetic variation of the fungus *Hymenoscyphus pseudoalbidus*. Doctoral Thesis (Faculty of Natural Resources and Agricultural Sciences, Uppsala) (2013).
2. Bakys, R. Dieback of *Fraxinus excelsior* in the Baltic sea region, associated fungi, their pathogenicity and implications for silviculture. Doctoral Thesis (Faculty of Natural Resources and Agricultural Sciences Uppsala) (2013).
3. Pautasso, M., Aas, G., Queloz, V. & Holdenrieder, O. European ash (*Fraxinus excelsior*) dieback - A conservation biology challenge. *Biol Cons* **158**, 37-49 (2013).
4. Zheng, H-D. & Zhuang, W-Y. *Hymenoscyphus albidoides* sp. nov. and *H. pseudoalbidus* from China. *Mycol Prog* **13**, 625-638 (2014).
5. Zhao, Y-J., Hosoya, T., Baral, H-O., Hosaka, K. & Kakishima, M. *Hymenoscyphus pseudoalbidus*, the correct name for *Lambertella albida* reported from Japan. *Mycotaxon* **122**, 25-41 (2013).
6. Han, J-G. *et al.* First report of the ash dieback pathogen *Hymenoscyphus fraxineus* in Korea. *Mycobiology* **42**, 391-396 (2014).
7. Cleary, M. *et al.* Friend or foe? Biological and ecological traits of the European ash dieback pathogen *Hymenoscyphus fraxineus* in its native environment. *Sci Rep* **6**, 21895, doi:10.1038/srep21895 (2016).
8. Harris, I., Jones, P.D., Osborn, T.J. & Lister, D.H. Updated high-resolution grids of monthly climatic observations - the CRU TS3.10 Dataset. *Int J Climatol* **34**, 623-642 (2013).
9. Ramírez-Villegas, J. & Bueno-Cabrera, A. Working with climate data and niche modeling: Creation of bioclimatic variables. Cali, Colombia, International Center for Tropical Agriculture (CIAT) (2009).
10. Roubicek, A.J. *et al*. Does the choice of climate baseline matter in ecological niche modelling? *Ecol Model* **221**, 2280-2286 (2010).
11. Bartlein, P.J., Prentice, I.C. & Webb, III T. Climatic response surfaces from pollen data for some eastern North American taxa. *J Biogeogr* **13**, 35-57 (1986).

12. Prentice, I.C. *et al*. Special paper: a global biome model based on plant physiology and dominance, soil properties and climate. *J Biogeogr* **19**, 117-134 (1992).
13. Pearman, P.B. *et al.* Prediction of plant species distributions across six millennia. *Ecol Lett* **11,** 357-369 (2008).
14. Thuiller, W., Lavorel, S., Araújo, M.B., Sykes, M.T. & Prentice, I.C. Climate change threats to plant diversity in Europe. *Proc Natl Acad Sci USA* **102**, 8245-8250 (2005).
15. Phillips, S.J., Anderson, R.P. & Schapire, R.E. Maximum entropy modeling of species geographic distributions. *Ecol Model* **190,** 231-259 (2006).
16. Merow, C., Smith, M.J. & Silander, J.A. A practical guide to MaxEnt for modeling species' distributions: what it does, and why inputs and settings matter. *Ecography* **36**, 1058-1069 (2013).
17. Elith, J. *et al*. A statistical explanation of MaxEnt for ecologists. *Divers Distrib* **17**, 43-57 (2011).
18. Phillips, S.J. & Dudík, M. Modeling of species distributions with Maxent: new extensions and a comprehensive evaluation. *Ecography* **31**, 161-175 (2008).
19. Radosavljevic, A. & Anderson, R.P. Making better Maxent models of species distributions: complexity, overfitting and evaluation. *J Biogeogr* **41**, 629-643 (2014).
20. Beaugrand, G., Lenoir, S., Ibañez, F. & Manté, C. A new model to assess the probability of occurrence of a species based on presence-only data. *Mar Ecol Prog Ser* **424**, 175-190 (2011).
21. Stockwell, D. The GARP modelling system: problems and solutions to automated spatial prediction. *Int J Geogr Inf Sci* **13**, 143-158 (1999).
22. Goberville, E., Beaugrand, G., Hautekèete, N-C., Piquot, Y. & Luczak, C. Uncertainties in the projection of species distributions related to general circulation models. *Ecol Evol* **5**, 1100-1116 (2015).
23. Mahalanobis, P.C. On the generalized distance in statistics. *Proc Nat Instit Sci India* **2**, 49-55 (1936).
24. Ibañez, F. Immediate detection of heterogeneities in continuous multivariate, oceanographic recordings. Application to time series analysis of changes in the bay of Villefranche sur Mer. *Limnol Oceanogr* **26**, 336-349 (1981).
25. Legendre, P. & Legendre, L. *Numerical Ecology* (Elsevier Science, Amsterdam) (2012).
26. Mielke, P.W., Berry, K.J. & Brier, G.W. Application of multi-response permutation procedures for examining seasonal changes in monthly mean sea-level pressure patterns. *Mon Weather Rev* **109**, 120-126 (1981).
27. Lenoir, S., Beaugrand, G. & Lécuyer, E. Modelled spatial distribution of marine fish and projected modifications in the North Atlantic Ocean. *Glob Change Biol* **17**, 115-129 (2011).
28. Raybaud, V. *et al.* Decline in kelp in West Europe and climate. *PLoS ONE* **8**, e66044 (2013).
29. Hanley, J.A. & McNeil, B.J. The meaning and use of the area under a receiver operating characteristic (ROC) curve. *Radiology* **143**, 29-36 (1982).
30. Allouche, O., Tsoar, A. & Kadmon, R. Assessing the accuracy of species distribution models: prevalence, kappa and the true skill statistic (TSS). *J Appl Ecol* **43**, 1223-1232 (2006).
31. Phillips, S.J. *et al*. Sample selection bias and presence-only distribution models: implications for background and pseudo-absence data. *Ecol Appl* **19**, 181-197 (2009).
32. Brotons, L., Thuiller, W., Araújo, M.B. & Hirzel, A.H. Presence-absence versus presence-only modelling methods for predicting bird habitat suitability. *Ecography* **27**, 437-448 (2004).
33. Huberty, C.J. *Applied discriminant analysis* (Wiley New York) (1994).
34. Lawson, C.R., Hodgson, J.A., Wilson, R.J. & Richards, S.A. Prevalence, thresholds and the performance of presence-absence models. *Methods Ecol Evol* **5**, 54-64 (2014).
35. Fithian, W., Elith, J., Hastie, T. & Keith, D.A. Bias correction in species distribution models: pooling survey and collection data for multiple species. *Methods Ecol Evol* **6**, 424-438 (2015).
36. Elith, J., Kearney, M. & Phillips, S. The art of modelling range-shifting species. *Methods Ecol Evol* **1**, 330-342 (2010).

37. Dormann, C.F. *et al.* Collinearity: a review of methods to deal with it and a simulation study evaluating their performance. *Ecography* **36**, 27-46 (2013).
38. Bucklin, D.N. *et al.* Comparing species distribution models constructed with different subsets of environmental predictors. *Divers Distrib* **21**, 23-35 (2015).
39. Pearson, R.G., Dawson, T.P. Predicting the impacts of climate change on the distribution of species: are bioclimate envelope models useful? *Global Ecol Biogeogr* **12**, 361-371 (2003).
40. Franklin, J. *et al*. Modeling plant species distributions under future climates: how fine scale do climate projections need to be? *Glob Change Biol* **19**, 473-483 (2013).
41. Hauptman, T. *et al*. Temperature effect on *Chalara fraxinea*: heat treatment of saplings as a possible disease control method. *Forest Pathol* **43**, 360-370 (2013).
42. Kowalski, T. & Bartnik, C. Morphologial variation in colonies of *Chalara fraxinea* isolated from ash (*Fraxinus excelsior* L.) stems with symptoms of dieback and effects of temperature on colony growth and structure. *Acta Agrobot* **63** (2010).
43. Pham, T.L.H., Zaspel, I., Schuemann, M., Stephanowitz, H. & Krause, E. Rapid in-vitro and in-vitro detection of *Chalara fraxinea* by means of mass spectrometric techniques. *Am J Plant Sci* **4**, 444-453, (2013).
44. Freeman, E.A., Moisen, G.G. A comparison of the performance of threshold criteria for binary classification in terms of predicted prevalence and kappa. *Ecol Model* **217**, 48-58 (2008).
45. Liu, C., Berry, P.M., Dawson, T.P. & Pearson, R.G. Selecting thresholds of occurrence in the prediction of species distributions. *Ecography* **28**, 385-393 (2005).
46. Liu, C., Newell, G. & White, M. On the selection of thresholds for predicting species occurrence with presence-only data. *Ecol Evol* **6**, 337-348 (2015).
47. Jiménez-Valverde, A. & Lobo, J.M. Threshold criteria for conversion of probability of species presence to either-or presence-absence. *Acta Oecol* **31**, 361-369 (2007).
48. Stocker, T.F. *et al*. Climate change 2013: The physical science basis. *Intergovernmental Panel on Climate Change, Working Group I Contribution to the IPCC Fifth Assessment Report (AR5)(Cambridge Univ Press, New York)* (2013).
49. Van Vuuren, D.P. *et al*. The representative concentration pathways: an overview. *Clim Change* **109**, 5-31 (2011).
50. Rogelj, J., Meinshausen, M. & Knutti, R. Global warming under old and new scenarios using IPCC climate sensitivity range estimates. *Nat Clim Chang* **2**, 248-253 (2012).
51. Wang, G.Q. *et al.* Assessing water resources in China using PRECIS projections and a VIC model. *Hydrol Earth Syst Sci* **16**, 231-240 (2012).
52. Sachindra, D.A., Huang, F., Barton, A. & Perera, B.J.C. Statistical downscaling of general circulation model outputs to precipitation-part 2: bias-correction and future projections. *Int J Climatol* **34**, 3282-3303 (2014).
53. Beaumont, L.J., Pitman, A.J., Poulsen, M. & Hughes, L. Where will species go? Incorporating new advances in climate modelling into projections of species distributions. *Glob Change Biol* **13**, 1368-1385 (2007).
54. Huntley, B., Green, R., Collingham, Y. & Willis, S. *A climatic atlas of European breeding birds* (Lynx Edicions) p528 (2008).
55. Ramírez-Villegas, J., Jarvis, A. Downscaling global circulation model outputs: The Delta method decision and policy analysis working paper No.1. Policy Analysis (2010).
56. Voldoire, A. *et al*. The CNRM-CM5. 1 global climate model: description and basic evaluation. *Clim Dynam* **40**, 2091-2121 (2013).
57. Syktus, J. *et al*. The CSIRO-QCCCE contribution to CMIP5 using the CSIRO Mk3.6 climate model (eds MODSIM) 2782-2788 (19th International Congress on Modelling and Simulation) (2011).
58. Dufresne, J-L. *et al*. Climate change projections using the IPSL-CM5 Earth System Model: from CMIP3 to CMIP5. *Clim Dynam* **40**, 2123-2165 (2012).
59. Jones, C.D. et al. The HadGEM2-ES implementation of CMIP5 centennial simulations. *Geosci Model Dev* **4**, 543-570 (2011).

60. Stevens, B. *et al*. Atmospheric component of the MPI-M earth System Model: ECHAM6. *J Adv Model Earth Sy* **5**, 146-172 (2013).
61. Shindell, D.T. *et al*. Radiative forcing in the ACCMIP historical and future climate simulations. *Atmos Chem Phys* **12**, 21105-21210 (2012).
62. Gent, P.R. *et al*. The community climate system model version 4. *J Climate* **24**, 4973-4991 (2011).

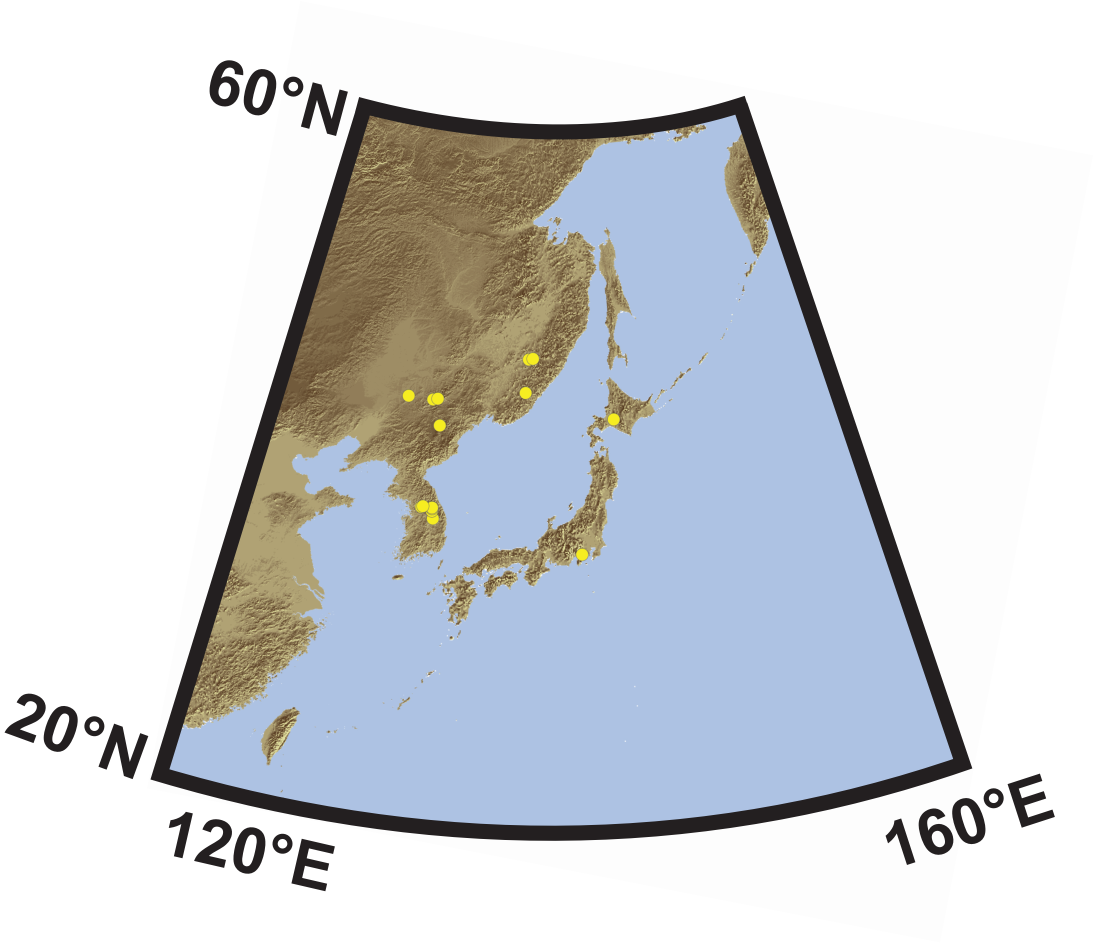

Supplementary Figure S1

a

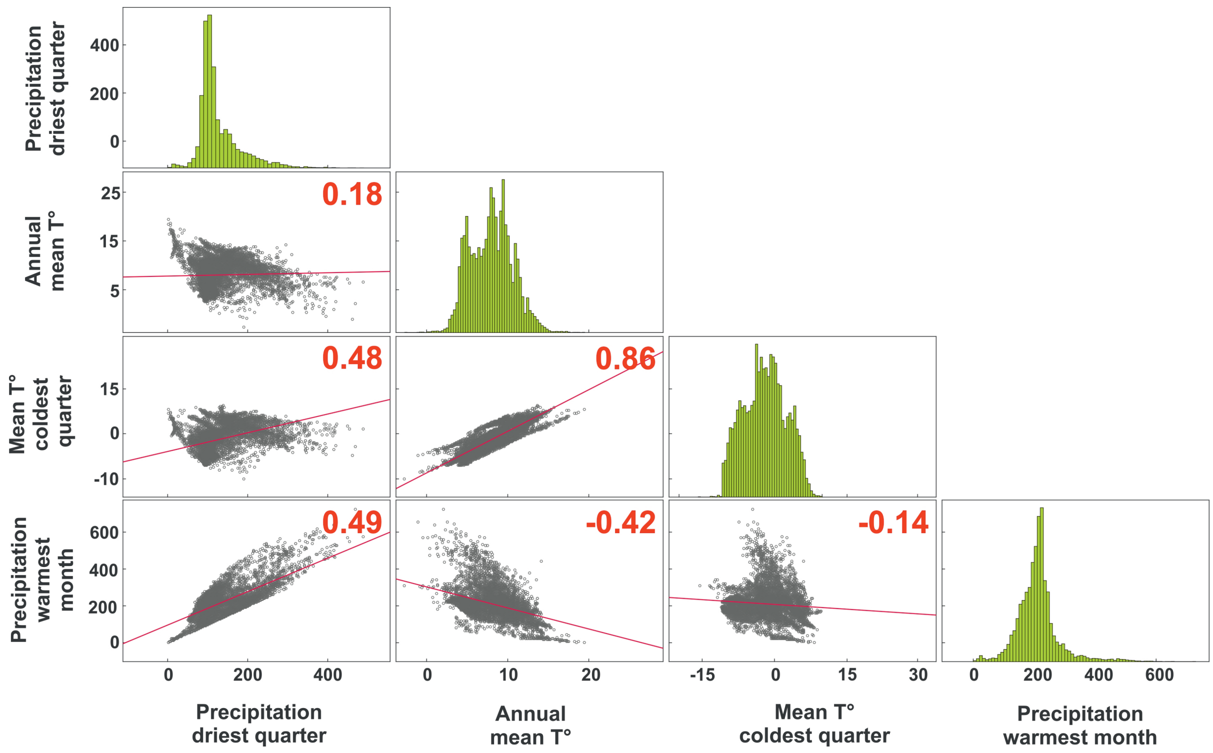

b

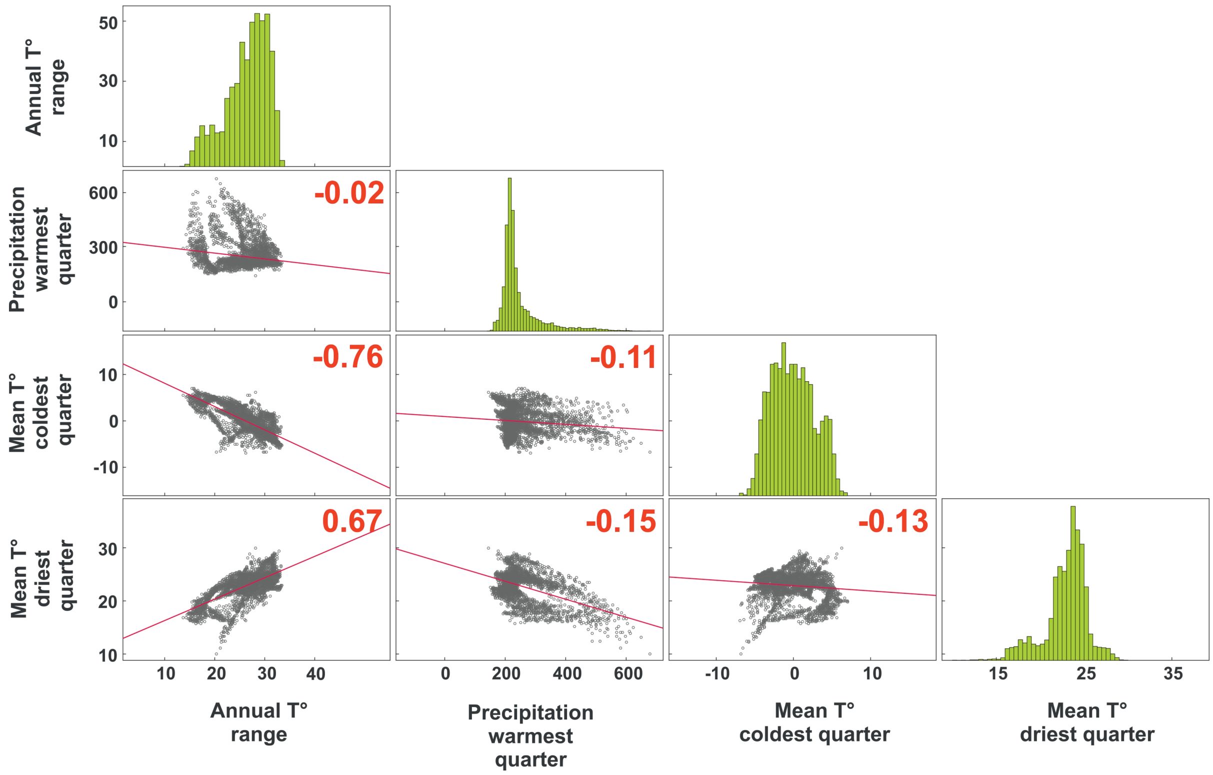

Supplementary Figure 2

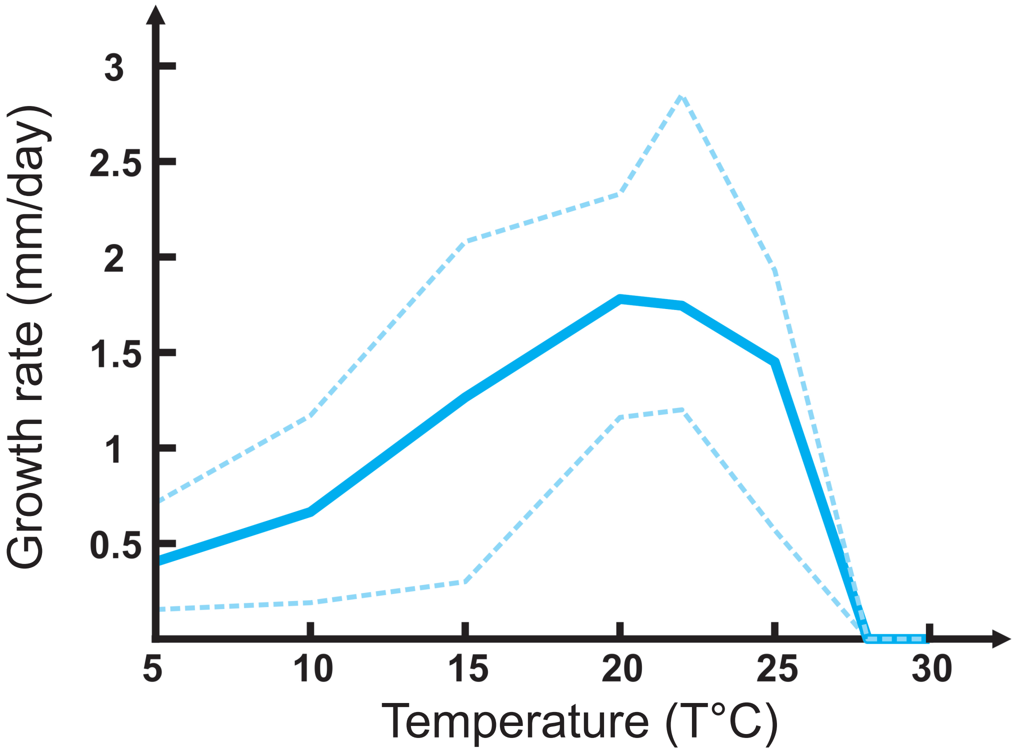

Supplementary Figure S3

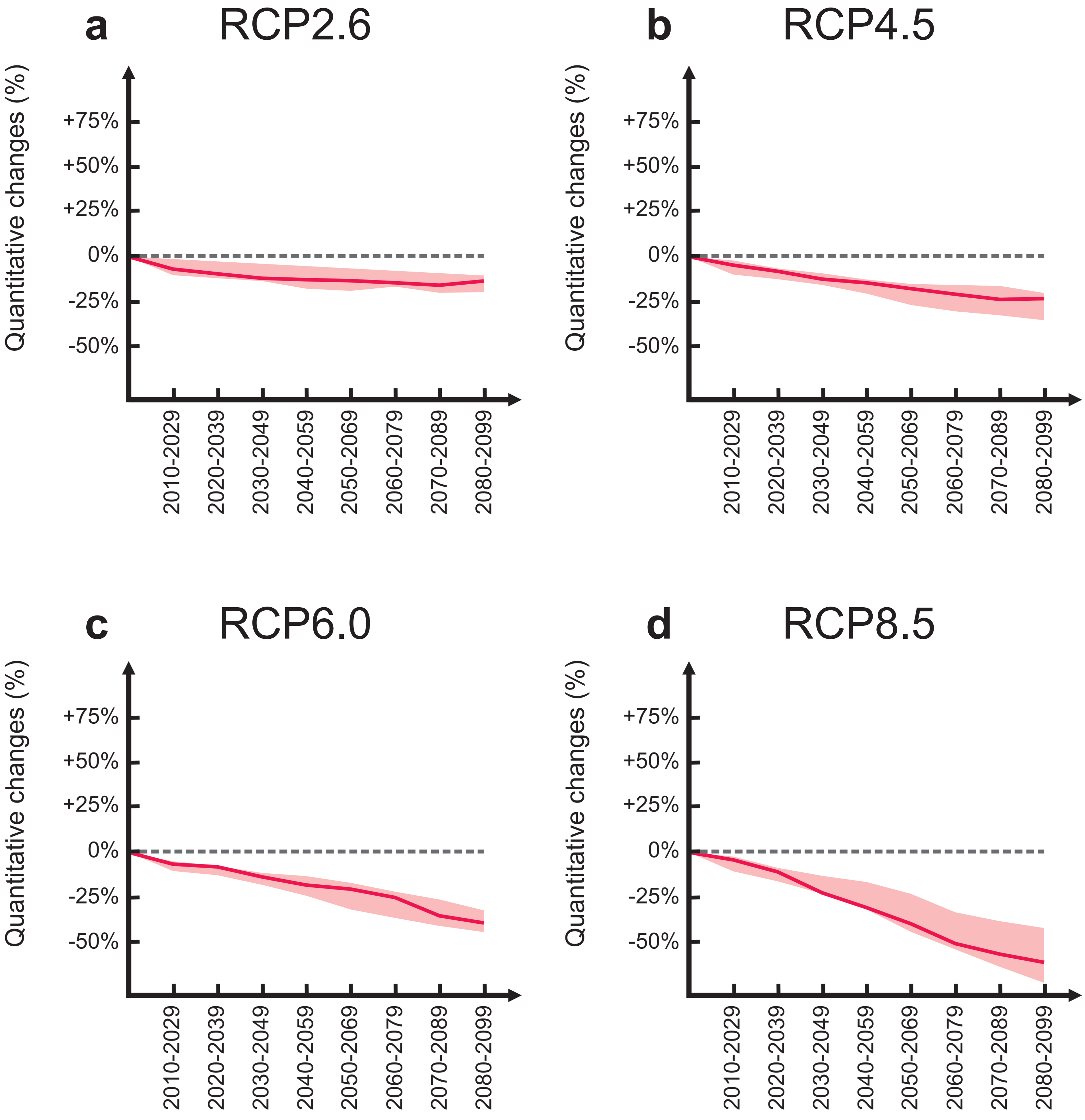

Supplementary Figure S4

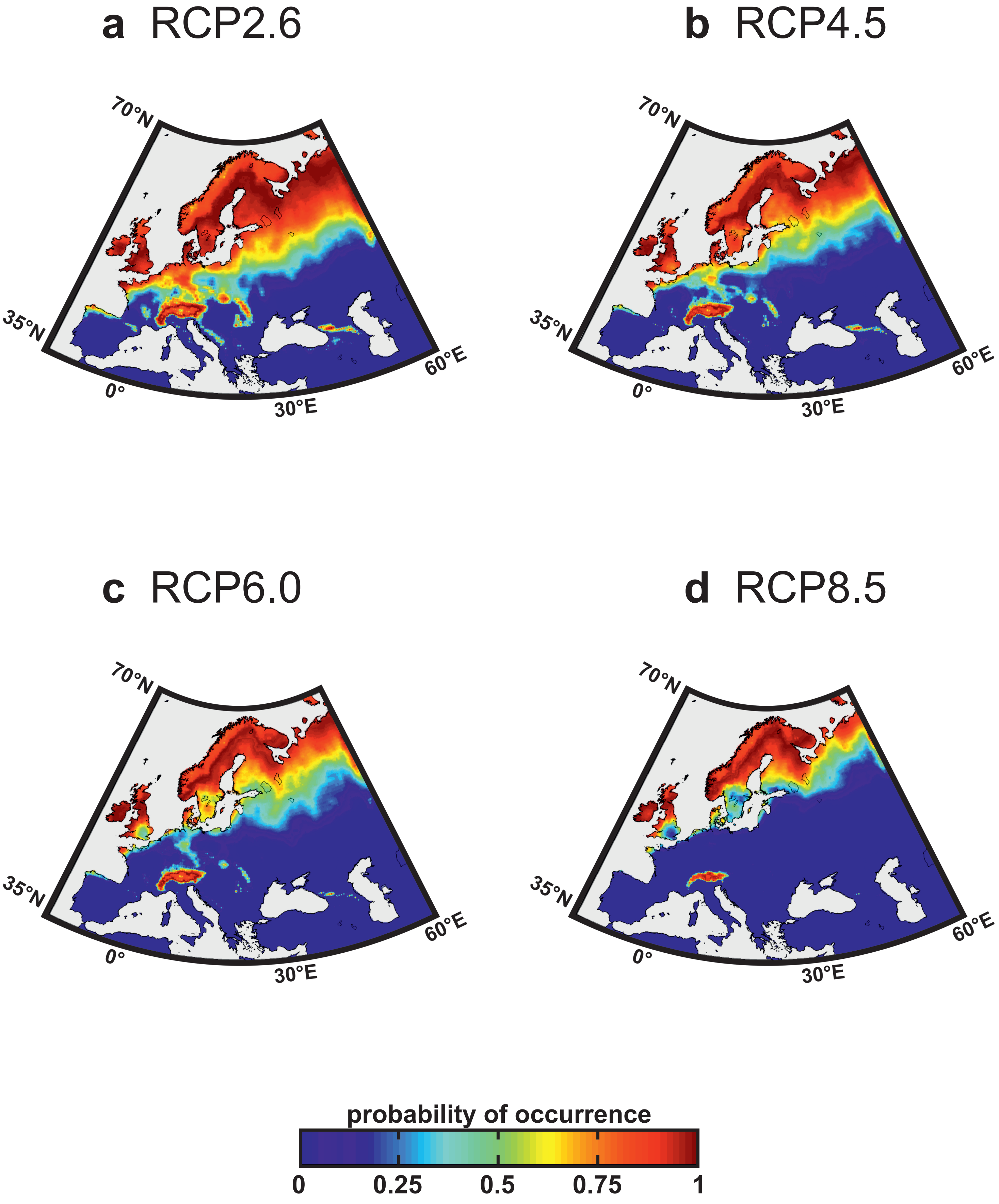

Supplementary Figure S5

a. Common ash

Bioclimatic parameters	Variable relative contribution	AUC	
**Precipitation of driest quarter**	**38.5**	**0.903**	***(1)***
**Annual mean temperature**	**28.8**	**0.971**	***(2)***
**Mean temperature of coldest quarter**	**15.3**	**0.977**	***(3)***
**Precipitation of warmest quarter**	**9.1**	**0.981**	***(4)***
Annual temperature range	2.9	0.986	*(5)*
Maximum temperature of warmest month	1.5	0.988	*(6)*
Precipitation of driest month	0.9	0.988	*(7)*
Mean temperature of driest quarter	0.6	0.988	*(8)*
Temperature seasonality	0.4	0.990	*(9)*
Temperature diurnal range	0.3	0.990	*(10)*
Precipitation of most humid month	0.3	0.991	*(11)*
Precipitation of coldest quarter	0.3	0.991	*(12)*
Mean temperature of warmest quarter	0.3	0.991	*(13)*
Isothermality	0.3	0.991	*(14)*
Annual precipitation	0.2	0.991	*(15)*
Precipitation seasonality	0.2	0.991	*(16)*
Precipitation of most humid quarter	0.1	0.991	*(17)*
Mean temperature of most humid quarter	0.1	0.992	*(18)*
Minimum temperature of coldest month	0	0.992	*(19)*

b. *H. fraxineus*

Bioclimatic parameters	Variable relative contribution	AUC	
**Annual temperature range**	**42**	**0.900**	***(1)***
**Precipitation of warmest quarter**	**20.2**	**0.971**	***(2)***
**Mean temperature of coldest quarter**	**13.1**	**0.982**	***(3)***
**Mean temperature of driest quarter**	**8.5**	**0.989**	***(4)***
Maximum temperature of warmest month	4.3	0.992	*(5)*
Minimum temperature of coldest month	2.7	0.992	*(6)*
Mean temperature of warmest quarter	2.5	0.992	*(7)*
Annual mean temperature	2.1	0.992	*(8)*
Isothermality	1.2	0.993	*(9)*
Temperature seasonality	1	0.994	*(10)*
Annual precipitation	0.9	0.997	*(11)*
Mean temperature of most humid quarter	0.4	0.997	*(12)*
Temperature diurnal range	0.3	0.998	*(13)*
Precipitation of driest month	0.2	0.998	*(14)*
Precipitation of coldest quarter	0.1	0.998	*(15)*
Precipitation of driest quarter	0.1	0.999	*(16)*
Precipitation seasonality	0	0.999	*(17)*
Precipitation of most humid quarter	0	0.999	*(18)*
Precipitation of most humid month	0	0.999	*(19)*

Supplementary Table S1

	Common ash	*H. fraxineus*
AUC		
**MaxEnt**	0.981 ± 0.001	0.989 ± 0.001
**NPPEN**	0.966 ± 0.001	0.975 ± 0.001
TSS		
**MaxEnt**	0.865 ± 0.003	0.919 ± 0.001
**NPPEN**	0.833 ± 0.003	0.826 ± 0.005
Threshold		
**MaxEnt**	0.270	0.150
**NPPEN**	0.330	0.100
Projected contemporary geographical extent (in million km^2)		
**MaxEnt**	7.32	3.71
**NPPEN**	7.01	3.42

Supplementary Table S2

Modeling Center	Institution	Model	Atmospheric resolution	Reference
CNRM - CERFAS	Centre National de Recherches Meteorologiques / Centre Europeen de Recherche et Formation Avancees en Calcul Scientifique	CNRM - CM5	1.4° x 1.4°	Ref. [56]
CSIRO - QCCCE	Commonwealth Scientific and Industrial Research Organisation / Queensland Climate Change Centre of Excellence	CSIRO - Mk3.6.0	1.87° x 1.87°	Ref. [57]
IPSL	Institut Pierre-Simon Laplace	IPSL - CM5A - LR	1.87° x 3.75°	Ref. [58]
MOHC	Met Office Hadley Centre	HadGEM2 - ES	1.87° x 1.25°	Ref. [59]
MPI - M	Max Planck Institute for Meteorology	MPI - ESM - LR	1.87° x 1.87	Ref. [60]
NASA - GISS	NASA Goddard Institute for Space Studies	GISS - E2 - R	2° x 2.5°	Ref. [61]
NCAR	National Center for Atmospheric Research	CCSM4	1.25° x 0.94°	Ref. [62]

Supplementary Table S3

a

Species	Period	RCP2.6	RCP4.5	RCP6.0	RCP8.5
		Quantitative changes expressed in percentage (%)			
**Common ash** (alone)	2010-2029	*-1.89* < **0.90** < *3.25*	*-2.36* < **-1.11** < *0.26*	*-1.87* < **0.38** < *1.60*	*-1.35* < **-0.51** < *1.10*
	2020-2039	*-1.62* < **1.93** < *4.59*	*0.17* < **2.19** < *4.31*	*-1.33* < **1.86** < *4.20*	*1.06* < **4.79** < *6.28*
	2030-2049	*2.98* < **4.18** < *7.72*	*4.62* < **5.78** < *9.23*	*-0.07* < **3.77** < *7.97*	*6.89* < **10.37** < *13.30*
	2040-2059	*3.84* < **5.60** < *8.38*	*7.15* < **7.55** < *12.67*	*3.26* < **5.26** < *14.85*	*10.31* < **12.46** < *19.03*
	2050-2069	*0.31* < **6.43** < *8.98*	*7.67* < **9.87** < *16.73*	*4.98* < **8.62** < *18.85*	*16.20* < **17.14** < *22.74*
	2060-2079	*1.86* < **7.23** < *10.68*	*9.57* < **11.30** < *19.07*	*4.65* < **11.03** < *19.96*	*21.30* < **22.93** < *24.54*
	2070-2089	*3.32* < **7.78** < *10.58*	*10.15* < **11.90** < *20.14*	*9.86* < **14.03** < *23.20*	*23.18* < **24.82** < *26.45*
	2080-2099	*3.35* < **7.05** < *12.49*	*10.66* < **14.21** < *21.43*	*14.39* < **16.58** < *24.51*	*24.55* < **26.59** < *26.95*
H. fraxineus (alone)	2010-2029	*-9.72* < **-8.78** < *-7.47*	*-10.13* < **-8.27** < *6.46*	*-4.71* < **1.97** < *4.78*	*-11.84* < **-8.70** < *3.01*
	2020-2039	*-15.42* < **-11.08** < *-2.66*	*-15.79* < **-11.79** < *5.23*	*-15.19* < **-4.80** < *4.10*	*-15.60* < **-10.04** < *5.07*
	2030-2049	*-13.67* < **-0.94** < *1.88*	*-11.34* < **-5.67** < *2.11*	*-18.20* < **-10.03** < *0.82*	*-19.13* < **0.77** < *8.43*
	2040-2059	*-17.26* < **-0.98** < *2.32*	*-12.90* < **-6.16** < *4.06*	*-17.35* < **-14.16** < *-2.96*	*-19.78* < **-4.25** < *6.45*
	2050-2069	*-11.42* < **-4.25** < *3.08*	*-19.50* < **-6.05** < *10.14*	*-21.90* < **-18.83** < *-5.71*	*-30.80* < **-6.32** < *8.03*
	2060-2079	*-7.77* < **-1.44** < *5.60*	*-26.22* < **-0.78** < *11.57*	*-24.04* < **-20.00** < *-8.94*	*-38.69* < **-9.85** < *7.49*
	2070-2089	*0.91* < **2.59** < *9.09*	*-29.91* < **5.77** < *6.28*	*-29.67* < **-19.10** < *-9.64*	*-46.34* < **-34.70** < *0.89*
	2080-2099	*-4.45* < **6.27** < *10.89*	*-32.18* < **-2.98** < *6.40*	*-34.29* < **-17.71** < *-2.46*	*-54.84* < **-47.51** < *-3.11*
**Common ash** (with *H. fraxineus*)	2010-2029	*11.10* < **13.81** < *19.60*	*-1.81* < **7.03** < *16.02*	*3.50* < **6.43** < *12.94*	*1.54* < **7.08** < *14.30*
	2020-2039	*14.20* < **16.75** < *26.38*	*10.66* < **15.61** < *24.45*	*10.91* < **12.99** < *19.72*	*17.83* < **21.70** < *25.74*
	2030-2049	*13.47* < **22.95** < *27.83*	*20.02* < **28.12** < *34.09*	*17.95* < **25.91** < *33.26*	*32.01* < **36.16** < *43.23*
	2040-2059	*21.83* < **27.84** < *33.11*	*23.07* < **34.08** < *40.74*	*31.58* < **37.62** < *46.61*	*39.86* < **44.34** < *55.99*
	2050-2069	*28.22* < **29.35** < *31.07*	*25.27* < **43.22** < *50.10*	*34.54* < **45.60** < *54.90*	*45.02* < **51.08** < *64.67*
	2060-2079	*27.40* < **31.23** < *33.12*	*29.14* < **44.26** < *53.31*	*35.58* < **51.18** < *61.66*	*51.51* < **64.43** < *72.03*
	2070-2089	*27.21* < **31.32** < *34.61*	*36.46* < **49.21** < *53.43*	*42.97* < **57.87** < *67.78*	*58.65* < **71.59** < *77.78*
	2080-2099	*22.03* < **29.46** < *32.51*	*38.20* < **54.92** < *57.57*	*52.30* < **62.64** < *69.53*	*63.70* < **70.69** < *83.10*

b

Species	Period	RCP2.6	RCP4.5	RCP6.0	RCP8.5
		Quantitative changes expressed in percentage (%)			
**Common ash** (alone)	2010-2029	*0.72* < **4.30** < *5.28*	*0.29* < **2.23** < *3.46*	*2.37* < **5.48** < *5.86*	*1.25* < **2.74** < *2.92*
	2020-2039	*2.53* < **5.11** < *7.05*	*3.11* < **5.99** < *7.32*	*2.65* < **5.90** < *6.70*	*4.24* < **7.74** < *9.71*
	2030-2049	*6.47* < **7.46** < *8.33*	*7.54* < **8.54** < *9.89*	*2.27* < **6.95** < *9.43*	*9.55* < **13.26** < *14.39*
	2040-2059	*7.07* < **8.08** < *10.11*	*9.26* < **9.51** < *12.64*	*5.62* < **7.30** < *16.12*	*12.02* < **13.73** < *17.05*
	2050-2069	*3.00* < **8.71** < *11.59*	*10.14* < **11.82** < *18.17*	*7.49* < **10.42** < *18.04*	*16.67* < **17.90** < *20.53*
	2060-2079	*4.45* < **10.36** < *13.22*	*12.34* < **13.24** < *19.20*	*6.79* < **12.69** < *15.66*	*16.64* < **19.88** < *22.11*
	2070-2089	*5.66* < **9.90** < *15.44*	*13.20* < **14.94** < *19.32*	*10.82* < **14.95** < *17.43*	*13.31* < **18.77** < *22.56*
	2080-2099	*5.80* < **9.38** < *16.00*	*14.34* < **16.59** < *19.62*	*14.91* < **15.91** < *18.07*	*7.83* < **18.28** < *21.86*
H. fraxineus (alone)	2010-2029	*-15.92* < **-13.96** < *-7.57*	*-13.26* < **-8.52** < *7.44*	*-9.36* < **-0.85** < *2.48*	*-15.28* < **-9.32** < *-2.10*
	2020-2039	*-17.79* < **-16.02** < *-10.70*	*-24.44* < **-13.39** < *2.68*	*-18.96* < **-5.62** < *-0.11*	*-21.21* < **-13.07** < *1.30*
	2030-2049	*-15.81* < **-7.25** < *-0.39*	*-19.08* < **-9.54** < *-6.38*	*-22.20* < **-18.25** < *-3.38*	*-28.61* < **-12.17** < *2.87*
	2040-2059	*-26.54* < **-7.41** < *0.43*	*-20.82* < **-6.12** < *-3.05*	*-26.13* < **-25.40** < *-8.81*	*-32.46* < **-21.14** < *0.91*
	2050-2069	*-20.54* < **-12.54** < *-2.01*	*-31.79* < **-7.48** < *5.87*	*-36.69* < **-31.44** < *-7.83*	*-40.82* < **-25.09** < *-0.15*
	2060-2079	*-16.57* < **-8.35** < *0.91*	*-35.95* < **-2.10** < *2.52*	*-36.53* < **-29.46** < *-11.67*	*-46.76* < **-23.46** < *-2.91*
	2070-2089	*-8.69* < **-3.10** < *0.40*	*-39.21* < **-3.02** < *2.37*	*-39.48* < **-35.93** < *-14.16*	*-50.97* < **-40.85** < *-6.93*
	2080-2099	*-10.42* < **-1.80** < *4.06*	*-40.27* < **-13.08** < *1.25*	*-42.12* < **-37.12** < *-16.31*	*-61.30* < **-47.08** < *-9.78*
**Common ash** (with *H. fraxineus*)	2010-2029	*11.29* < **15.05** < *17.76*	*2.77* < **6.10** < *13.66*	*7.12* < **11.25** < *13.90*	*5.77* < **11.22** < *13.21*
	2020-2039	*10.64* < **16.11** < *22.75*	*11.56* < **12.84** < *18.18*	*10.10* < **13.69** < *19.48*	*13.99* < **19.21** < *23.46*
	2030-2049	*14.14* < **16.35** < *20.98*	*18.16* < **20.31** < *23.55*	*12.95* < **19.64** < *24.24*	*22.29* < **25.44** < *31.57*
	2040-2059	*16.17* < **23.30** < *23.45*	*21.82* < **24.39** < *26.84*	*23.03* < **26.39** < *30.79*	*29.31* < **33.36** < *36.20*
	2050-2069	*17.20* < **22.25** < *26.80*	*23.61* < **27.56** < *34.45*	*25.99* < **32.18** < *36.06*	*36.28* < **43.00** < *45.17*
	2060-2079	*18.60* < **23.40** < *27.30*	*26.55* < **33.00** < *39.44*	*25.59* < **32.21** < *39.44*	*38.33* < **47.39** < *51.34*
	2070-2089	*16.31* < **24.64** < *28.83*	*31.67* < **34.16** < *39.08*	*32.30* < **39.31** < *43.89*	*43.86* < **46.83** < *48.99*
	2080-2099	*16.67* < **21.03** < *30.01*	*29.92* < **35.36** < *42.89*	*37.04* < **38.84** < *47.72*	*41.90* < **44.59** < *47.41*

Supplementary Table S4
